# The Subcutaneous Implantable Cardioverter-Defibrillator: A Patient Perspective

**DOI:** 10.3390/jcm12206675

**Published:** 2023-10-22

**Authors:** Martina Nesti, Vincenzo Russo, Zefferino Palamà, Luca Panchetti, Silvia Garibaldi, Umberto Startari, Gianluca Mirizzi, Marcello Piacenti, Andrea Rossi, Luigi Sciarra

**Affiliations:** 1 Fondazione Toscana Gabriele Monasterio, 56124 Pisa, Italy; lucapanchett@ftgm.it (L.P.); sgaribaldi@ftgm.it (S.G.); umbes@ftgm.it (U.S.); gianluca.mirizzi@ftgm.it (G.M.); piacenti@ftgm.it (M.P.); rossi79@ftgm.it (A.R.); 2 Cardiology Unit, Department of Medical Translational Sciences, University of Campania “Luigi Vanvitelli”—Monaldi Hospital, 80126 Naples, Italy; v.p.russo@libero.it; 3 Electrophysiology Service, Division of Cardiology, Casa di Cura Villa Verde, 74121 Taranto, Italy; zefferino.palama@icloud.com; 4 Department of Cardiology (UTIC), Università degli Studi dell’Aquila, 67100 L’Aquila, Italy; lui.sciarra@gmail.com

**Keywords:** S-ICD, anxiety, professional life, distress

## Abstract

The subcutaneous implantable cardioverter-defibrillator (S-ICD) is a new technology for the management of ICD patients. But what is the patients’ perspective? Previous studies on the transvenous ICD (TV-ICD) showed that device implantation is related not only to anxiety and depression because of the fear of ICD shocks, but also to many biopsychosocial factors like body image changes, perceived reduction of socialization and limitation in professional and sports activities. Anxiety and distress are more evident in younger women because of aesthetic reasons. The scar size and the position of the S-ICD can help these patients and positively influence their social relationships. Moreover, the position of the S-ICD reduces possible complications from catheters due to stress injury and can improve patients’ professional life by avoiding some work activity limitations. An S-ICD can be also a good option for athletes in avoiding subclavian crash and reducing inappropriate shocks. However, some questions remain unsolved because an S-ICD is not suitable for patients with indications for pacing, cardiac resynchronization therapy or anti-tachycardia pacing. In conclusion, the use of an S-ICD can assist physicians in reducing the negative impact of implantation on the well-being of some groups of patients by helping them to avoid depression and anxiety as well as improving their noncompliance with their medical treatment.

## 1. Introduction

The subcutaneous implantable cardioverter-defibrillator (S-ICD) is an established therapy for the prevention of sudden cardiac death (SCD) and an alternative to a transvenous ICD in selected patients. In the clinical practice, an S-ICD is indicated when pacing therapy for bradycardia support, cardiac resynchronization or anti-tachycardia pacing is not needed, or when venous access is difficult after the removal of a transvenous ICD for infections or in young patients with a long-term need for ICD therapy [1,2].

Cardiac device implantations can negatively impact patients’ psychological health; several studies showed that the fear of ICD shocks or complications is not the only cause of the high prevalence of generalized anxiety and depression [3]. Despite increasing data about the clinical safety and effectiveness of S-ICD system in prevention of SCD [4,5,6,7,8], little is known about the patient’s perspective. The aim of our review is to describe the patient’s perspective on S-ICD-related issues.

### S-ICD Patients’ Acceptance

The device patient’s acceptance is defined as the psychological accommodation and understanding of the device’s risk/benefit ratio, the recommendation of the device to others and the derivation of benefit in terms of biomedical, psychological and social functioning. In a multicenter Italian study of 176 patients with an S-ICD, Vicentini et al. [9] showed a positive acceptance of the S-ICD, particularly among young patients, as evaluated by the Florida Acceptance Survey at 12-month follow-up. This result was confirmed even in groups at risk of more distress such as women or patients with a body mass index (BMI) ≤ 25, and regardless of the generator’s positioning. Among patients receiving ICDs for HFrEF, the S-ICD was associated with better patient acceptance versus the transvenous ICD (TV-ICD).

The embarrassment due to an altered body image has an impact on patients’ social relationships. Even if it is often only the perception of the patient and not related to the real severity of the disfigurement [10], the scar size and the position of the implanted device can help this category of patients to accept the device. On the contrary, however, the TV-ICD can result in body image discomfort for some patients due to the bump under the skin in the subclavian region [11].

Body image concerns should be discussed with the patient before implantation, sharing with them the decision about which ICD is preferable. Good communication between a patient and physician plays a crucial role for the patient’s satisfaction with treatment [12], particularly during the decision-making process. It is crucial to avoid a negative impact of the implant on the well-being of patients to reduce depression and anxiety that could increase the risk of ventricular arrhythmias (VAs) and mortality [13], as well as causing general noncompliance with medical treatment [14].

## 2. Quality of Life and Stress

The experience of ICD implantation and the following acceptation of the device is not always easy and can cause psycho-physical distress. Only three studies have evaluated the impact of an S-ICD versus a TV-ICD on quality of life and stress [15,16,17]. A case–control study revealed a better physical well-being of S-ICD patients with a similar mental well-being compared to those with a TV-ICD [15]. In the EFFORTLESS registry and in the MIDAS study, no statistically significant differences in physical and mental quality of life and depression were found between S-ICD and TV-ICD patients at baseline and during a follow-up at 12 months [16]. The S-ICD patients experienced reductions in anxiety and greater reductions in depressive symptoms [17]. The authors hypothesized that the reduced invasiveness perceived by S-ICD patients was related to the absence of intravascular leads. Moreover, the smaller size and weight of the second-generation S-ICD may have improved the acceptance of the system. El-Battrawy et al. showed that 3.5% of short QT syndrome patients who were implanted with a TV-ICD experienced psychological distress at a 1-year follow-up, which was different from those with an S-ICD [18]. 

Ingles et al. [19] showed that higher anxiety scores among ICD patients were associated with female gender, syncope, other comorbidity and previous ICD-related complications (infection, lead fractures and device therapies). Regarding the gender differences, female patients who underwent ICD implantation were considered more susceptible to anxiety and psychosocial distress, due to several biopsychosocial factors such as body image changes and perceived reductions in femininity and socialization [20,21]. Younger women patients are at risk of future psychological and quality-of-life problems [22].

## 3. Work Activity

Few and contrasting data are available about the impact of an S-ICD on work activities. In a study of 42 patients who underwent S-ICD implantation, the limitations in professional life were graded relatively low [15] as compared to other studies in which over one-third of patients reported a negative influence of the device on their career [23]. To decrease lead-related complications, an S-ICD could be a good choice for patients with daily work activities that place them at increased risk of continuous or repetitive stress injuries (police officers or manual laborers).

## 4. Physical Activity

Physical activity is recommended in healthy subjects to prevent cardiovascular diseases [24,25] and in patients with cardiovascular diseases to reduce the residual cardiovascular risk [26]. Physical activity is considered a therapy that needs to be tailored according to the patient’s history, in order to balance benefits and potential adverse events. A large number of patients with cardiovascular disease are ICD recipients [27]. Among them, the ICD-related adverse events during physical activity are due to physical trauma, with consequent lead dislocation or fracture; myopotentials leading to inappropriate ICD shock; inappropriate shocks due to sinus tachycardia and dizziness; or pre-syncope caused by latency between the onset of arrhythmia and ICD intervention [28].

A large multicenter prospective registry [29] demonstrated the safety of high-risk athletes with ICDs in competitive sports. Nevertheless, data on the natural history of the cardiac diseases and on the efficacy of an ICD in terminating life-threatening arrhythmias during intense exercise are lacking and resulted in the restrictive nature of the guidelines for sports in ICD patients [30]. International guidelines recommend only moderate, leisure-time physical activity in patients with an ICD. For this reason, ICD athletes become ineligible for many competitive sports and can practice only low-intensity sports (billiards, bowling, cricket, curling, golf and riflery). But, these recommendations do not evaluate the differences between a TV-ICD and S-ICD [31]. 

One of main reasons that may drive the choice between a TV-ICD and S-ICD is the likelihood of injury to the lead or generator from a specific sport. Lead malfunction is one of the major issues for the ICD system. In an unselected population, the lead survival free of malfunction ranged from 85 to 98% at 5 years [32].

According to the ICD Sports Safety Registry [33], lead survival free of malfunction was 93% at 5 years, but the entrapment and compression of the lead between the clavicle and first rib, the so-called “subclavian crush syndrome”, is a well-known cause of TV-ICD lead malfunction [34]. It was hypothesized that sports with intensive involvement of the arms, such as swimming, may increase the subclavian crush. Even if long-term follow-up data on S-ICD leads are not available, athletes wishing to participate in these sports should avoid the use of a TV-ICD in order to reduce the risk of subclavian syndrome. In contrast, for contact sports, such as American football or ice hockey, the TV-ICD should be preferred since the generator is protected by standard padding.

## 5. Impact of ICD Therapies

S-ICD patients who experienced device therapies showed higher device-related distress [9]. This finding is consistent with that of previous evidence regarding the TV-ICD. The ICD shocks negatively impacted quality of life [35] and were related to higher levels of anxiety [36]. 

## 6. S-ICD Infection

Infections of cardiac implantable electronic devices (CIED) are an important complication and problem for cardiologists [37], ranging from 0.6 to 3.41% yearly [38,39,40,41,42,43]. CIED infections are related to higher mortality and renal failure [44].

In the prospective, multicenter IDE study [45], which included 330 patients who underwent S-ICD implantation, only 18 showed suspected or confirmed infections and only 4 of them required device explant. In 14 patients (4.4%), superficial or incisional infections were managed without system explant, by antibiotic therapy (n = 13) or sternal wound revision (n = 1). 

In the EFFORTLESS study [6] which included nearly 1000 S-ICD patients, the most common device-related complications were infections requiring device removal (2.4%), followed by erosion (1.7%).

A secondary analysis of the PRAETORIAN study showed that S-ICDs were associated with lower incidences of infections than transvenous devices, with a rate ranging from 0.25 to 1.1% yearly [46,47,48].

Recently, Gasperetti et al. [49] showed an overall incidence of infection of 9.3% among a large, real-world multicenter S-ICD cohort. Among these patients, 76.4% required reintervention. Similar to the TV-ICD, chronic kidney disease, higher body mass index and the presence of pocket hematoma were good predictors of such complications.

In a cohort of patients with malfunctioning leads who underwent implantation of a TV-ICD with extraction or an S-ICD without extraction of leads, there was no significant increase in adverse outcomes for patients who did not require pacing [50]. These data confirmed that the implantation of an S-ICD after abandonment of leads represents an alternative approach in particular settings (e.g., patients at high-risk, failed extractions, etc.) to reduce the incidence of complications due to extraction.

### 6.1. Suboptimal Pulse Generator or Electrode Position 

The implantation of an S-ICD system avoids many of the complications associated with transvenous leads, such as pneumothorax, cardiac tamponade and bacteremia, by placing all the metalware outside of the thorax. However, the pulse generator position can create discomfort or pain, but the intermuscular positioning during the revision of the system can solve these symptoms. On the other hand, lead-related complications are very rare; in December 2020, a medical advisory letter regarding EMBLEM S-ICD electrode model 3501 (Boston Scientific, Marlborough, MA, USA) was released by Boston Scientific Corporation [51] describing 27 cases of electrode body fractures, resulting in life-threatening events of 1 in 25,000 at 10 years. The use of remote monitoring may help physicians to check the systems and reduce the incidence of such complications.

### 6.2. S-ICD and Inappropriate Shocks

Early European and American studies of S-ICD patients, such as the EFFORTLESS (Evaluation of Factors Impacting Clinical Outcome and Cost Effectiveness of the S-ICD) [6,52] and IDE (Investigational Device Exemption) trials [45], showed a relatively high rate of inappropriate shocks (IASs) (1-year rates were 13.1% for IDE and 8.1% for EFFORTLESS). Most recently, the ELISIR study confirmed this trend with a rate of 8.9% [50]. The IASs were mainly due to T-wave oversensing and the failure to activate the conditional zone of the device where the discrimination algorithms are used [53]. The first step in reducing IASs was to program high-rate cutoffs for discrimination. More recently, the algorithm was modified to further reduce the risk of oversensing [54]. The latest improvement, added to the Gen 3 device, was the SMART Pass filter, which reduced the 1-year IAS rates by 50% in a real-world population [55,56]. The efficacy of these features was confirmed by the UNTOUCHED study: the 1-year rate of IASs was 3.1%, which was further reduced to 2.4% when only considering the Gen 3 devices [5]. The PRAETORIAN study showed that the S-ICD was noninferior to the TV-ICD regarding the incidence of IASs. This result was even more relevant because the SMART Pass filter was unavailable or not activated in the majority (78%) of patients [1].

The incidence of IASs differs according to different cardiomyopathies. Patients with ischemic cardiomyopathy showed a high incidence of IASs (3.6%/year vs. 1.7%/year, *p* = 0.048) compared to those with non-ischemic cardiomyopathy [57]. Patients with hypertrophic cardiomyopathy showed frequent IASs (3.8–6.9 per 100 patient years) [58,59]. The most frequent causes of IASs were sinus tachycardia (often related to the young age of the patients, heart failure or low compliance to the drug therapy), supraventricular arrhythmia, tall R waves and T-wave oversensing. 

In a study of 39 BrS patients who underwent S-ICD implantation, at the 2.5 year follow-up, 18% suffered IASs, mainly because of T-wave oversensing. The only independent predictor of the incidence of IASs was a younger age at diagnosis [60]. In a real-world setting of drug-induced type-1 BrS patients with ICD, no significant differences in inappropriate ICD therapies, device-related complications or infections were shown among S-ICD vs. TV-ICD patients [61,62]. 

In cardiomyopathies and channelopathies, ventricular repolarization abnormalities can change during tachycardia because of aberrant frequency-depent, which can compromise the S-ICD detection. In this setting, it is of pivotal importance to perform ECG screening both at rest and during exercise [63,64]. S-ICD system migration, often due to patients’ changes in weight or incorrect anchoring of the electrocatheter sleeve, may be responsible for IASs [65]. Here, a system revision is the correct management for reducing the risk of IASs and their consequences [66]. Subcutaneous air entrapment has been recently considered an underdiagnosed cause of early postimplant IASs, accounting for a part of oversensing of low amplitude signals [67]. Keeping the tissue moist via flushing with sterile saline, massaging the skin along the electrode and removing any residual subcutaneous air out through the incisions prior to closing are necessary to avoid this kind of complication. A lateral chest X-ray should be considered after implantation to reveal air surrounding the proximal electrode.

## 7. Conclusions

From a patient’s perspective, ICD implantation is a stressful and traumatic event leading to potential limitations in daily, work and sport activities. The S-ICD is a well-known therapy for the prevention of sudden cardiac death and represents an alternative to a TV-ICD system in selected patients. However, based on the available evidence, the choice to implant S-ICD 225 should be based on the patient’s tailored approach which takes into account the adequate S-ICD screening at rest or during stress, the nature of the underlying disease, the potential mechanisms of ventricular arrhythmias, the infective risk, the work or sportive activity and the psychosocial issues. 

## 8. Future Directions

Strong efforts are needed to reduce the psychological impact of S-ICD implantation. Reducing the device dimensions and increasing the battery longevity are some of the strategies for increasing patient acceptance. 
